# Ebolavirus diagnosis made simple, comparable and faster than molecular detection methods: preparing for the future

**DOI:** 10.1186/s12985-018-0985-8

**Published:** 2018-04-23

**Authors:** Ameh S. James, Shawn Todd, Nina M. Pollak, Glenn A. Marsh, Joanne Macdonald

**Affiliations:** 1 Genecology Research Centre, School of Science and Engineering, University of the Sunshine Coast, Sippy Downs, QLD Australia; 2 Inflammation and Healing Research Cluster, School of Science and Engineering, University of the Sunshine Coast, Sippy Downs, QLD Australia; 3 CSIRO Health and Biosecurity, Australian Animal Health Laboratory, Geelong, Australia; 4 Division of Experimental Therapeutics, Department of Medicine, Columbia University, New York, USA

**Keywords:** Ebolavirus, Molecular diagnostic, Rapid assay, Isothermal amplification technology, Low Resource Laboratories

## Abstract

**Background:**

The 2014/2015 Ebolavirus outbreak resulted in more than 28,000 cases and 11,323 reported deaths, as of March 2016. Domestic transmission of the Guinea strain associated with the outbreak occurred mainly in six African countries, and international transmission was reported in four countries. Outbreak management was limited by the inability to rapidly diagnose infected cases. A further fifteen countries in Africa are predicted to be at risk of Ebolavirus outbreaks in the future as a consequence of climate change and urbanization. Early detection of cases and reduction of transmission rates is critical to prevent and manage future severe outbreaks. We designed a rapid assay for detection of Ebolavirus using recombinase polymerase amplification, a rapid isothermal amplification technology that can be combined with portable lateral flow detection technology. The developed rapid assay operates in 30 min and was comparable with real-time TaqMan™ PCR.

**Methods:**

Designed, screened, selected and optimized oligonucleotides using the NP coding region from Ebola Zaire virus (Guinea strain). We determined the analytical sensitivity of our Ebola rapid molecular test by testing selected primers and probe with tenfold serial dilutions (1.34 × 10^10−^ 1.34 × 10^1^ copies/μL) of cloned NP gene from Mayinga strain of *Zaire ebolavirus* in pCAGGS vector, and serially diluted cultured Ebolavirus as established by real-time TaqMan™ PCR that was performed using ABI7500 in Fast Mode. We tested extracted and reverse transcribed RNA from cultured *Zaire ebolavirus* strains – Mayinga, Gueckedou C05, Gueckedou C07, Makona, Kissidougou and Kiwit. We determined the analytical specificity of our assay with related viruses: Marburg, Ebola Reston and Ebola Sudan. We further tested for Dengue virus 1–4, *Plasmodium falciparum* and West Nile Virus (Kunjin strain).

**Results:**

The assay had a detection limit of 134 copies per μL of plasmid containing the NP gene of Ebolavirus Mayinga, and cultured Ebolavirus and was highly specific for the *Zaire ebolavirus* species, including the Guinea strain responsible for the 2014/2015 outbreak. The assay did not detect related viruses like Marburg, Reston, or Sudan viruses, and other pathogens likely to be isolated from clinical samples.

**Conclusions:**

Our assay could be suitable for implementation in district and primary health laboratories, as only a heating block and centrifuge is required for operation. The technique could provide a pathway for rapid screening of patients and animals for improved management of outbreaks.

## Background

The Ebolavirus outbreak dominated international news in 2014 and the World Health Organization reported more than 28,000 cases worldwide and over 11,000 deaths [1]. After the initial outbreak in Guinea in December 2013 [2], there was intense and wide-spread transmission to neighbouring countries including Sierra Leone and Liberia. The virus was also exported to Mali, Nigeria, Senegal, the United States of America, and Europe [3, 4] Transmission was drastically reduced after global intervention, and all the affected countries have been declared “Ebola free” and travel restrictions have been lifted [5]. Nonetheless, 15 countries in Africa are predicted to be at risk of Ebolavirus outbreaks [6], based on the proximity of people to animals that harbour the virus, as well as environmental factors including climate change and urbanization. The Freetown Declaration on the 16th of October 2015 at the end of the Ebola outbreak in Sierra Leone called for action against the re-emergence of Ebolavirus, including the improvement of laboratory facilities for early detection [7]. The most recent reported Ebolavirus disease (EVD) outbreak, in July 2017 in the Democratic Republic of Congo, with 5 laboratory confirmed cases and 4 deaths [8] further stresses the importance of action in the diagnostic space.

Early laboratory diagnosis is pivotal for the prevention of re-emergence of Ebolavirus. Current laboratory diagnosis includes real-time polymerase chain reaction (RT-PCR), antigen-capture enzyme-linked immunosorbent assay (ELISA), immunoglobulin M (IgM) and immunoglobulin G (IgG) ELISA [9–16]. Additionally, there have been recent Ebolavirus assay developments using antibody and antigen detection and isothermal amplification technologies [17–20]. The assays deployed during the last Ebola outbreak were a combination of antigen-antibody-based and nucleic acid tests: RealStar Zaire Ebolavirus RT-PCR Kit (Altona Diagnostics, Hamburg, Germany); GeneXpert® Ebola Assay (Cepheid, Sunnyvale, California, USA), FilmArray® BioThreat/Ebola Panels (BioFire, Salt Lake City, Utah, USA); ReEBOV Antigen Rapid Test (Corgenix, Broomfield, Colorado, USA); SD Q Line Ebola Zaire Ag test (SD Biosensor, Suwon, Gyeonggi-do, Republic of Korea). The deployment of these assays depended on laboratory capabilities, rapid turn-around and easy use of the Ebolavirus test device. However, these assays did not consider any future detection of the virus; a future Ebolavirus outbreak has been predicted to affect 15 countries in Africa [6]. Additionally, the molecular assay results are not compatible with clinical laboratories that lack PCR expertise and infrastructural requirements, making the assays less relevant to technicians in resource-limited settings. Detection of viral RNA has proven to be most effective for diagnosis of Ebolavirus infections from the early to late stage of illness, however the standard RT-PCR Ebola assay is not readily available in areas where it is most needed. Among the isothermal technologies developed [21–30], recombinase polymerase amplification (RPA) appears very amenable to resource limited settings [28, 31]. The technology is fast, amplifying nucleic acids in 10 min, and can operate at body temperature or even lower (22 °C to 38 °C) [32]. The sensitivity and specificity of RPA is comparable to PCR, and is amenable to all PCR-derived amplification detection strategies including, real-time fluorescence and lateral flow strips (LFS) [31]. RPA has previously been reported for the detection of several RNA viruses, including Middle East respiratory syndrome coronavirus, Rift Valley fever virus, Ebolavirus and several filoviruses. These viruses were detected using portable fluorescent equipment, a real-time procedure [32–40]. Here we describe a rapid, sensitive, and specific assay for *Zaire ebolavirus*, which includes the current Guinea strain. Our assay uses RPA, but rather than using real-time fluorescent imaging, the assay uses the LFS as a detection format, making results very simple to interpret. A similar approach has also been used in the detection of RNA viruses such as Yellow fever virus [41] and *Chlamydia trachomatis* diagnosis [42]. The LFS component of the test (Milenia Biotec, Giessen, Germany) is designed to detect an RPA amplicon dual-labelled with FAM and biotin (supplied in the RPA primers and probes and incorporated during the amplification step). The amplicon is captured by gold-nanoparticles labelled with FAM-specific antibodies in the sample application area, before traversing to immobilized anti-biotin antibodies bound at a test line; precipitation of the gold nanoparticles at the test line results in the appearance of a red-blue band. Excess gold nanoparticles are also captured by species-specific antibodies bound to a control line; appearance of a red-blue band in the control line confirms correct operation of the strips in the absence of dual-labelled amplicon.

Our assay using the RPA-LFS method is highly amenable for low-resource laboratory, and has the potential to be deployed in future Ebolavirus outbreaks. The aim of this study was to develop an assay that is comparable and faster than RT-PCR with easy to interpret results. Additionally, we sought an assay that could be easily deployed in an outbreak situation, where limited resources preclude PCR laboratory facilities.

## Methods

### Sample preparation

Ebolavirus strains and related viruses (Table 1) were grown on Vero E6 cells and harvested from infected cell culture supernatant after centrifugation at 12,000 g. Stocks determined to have > 10^6^ PFU/mL by standard plaque assay were used for RNA extraction (140 μL) using an RNeasy Mini Kit (QIAGEN, Australia) according to the manufacturer’s instructions. All the procedures and manipulation of Ebolavirus-infected cultures were performed in a Biosafety Level 4 Laboratory at the CSIRO Australian Animal Health Laboratory. All samples were eluted in 50 μL nuclease free water, and 5 μL was used in a 20 μL reverse transcription reaction. Reverse transcription was performed using SuperScript IV Reverse Transcriptase (Thermo Fisher Scientific, MA, USA) and random hexamers (Thermo Fisher Scientific, MA, USA) according to manufacturer’s instructions, and 5 μL resultant cDNA was used as template for RPA-LFS. Dengue 1–4 and West Nile virus (Kunjin NSW 2011 strain) RNA extractions were a gift from Professor Roy Hall (University of Queensland, Brisbane, QLD, Australia) and *Plasmodium falciparum* (malaria) genomic DNA was a gift from Prof James McCarthy (QIMR-Berghofer, Brisbane, QLD, Australia).

**Table 1:** Ebolavirus strains and related viruses used for RT-RPA-LFS assay

Filovirus Species	Ebolavirus strain	Accession Number	Origin	Date
*Zaire ebolavirus*	Mayinga	AF086833.2	Zaire	1976
*Zaire ebolavirus*	Kikwit	KR867676.1	Zaire	1995
*Zaire ebolavirus*	Kissidougou	KJ660346.1	Guinea	2014
*Zaire ebolavirus*	Makona	KJ660347.2	Guinea	2014
*Zaire ebolavirus*	Makona-Gueckedou C05	KJ660348.1	Guinea	2014
*Zaire ebolavirus*	Gueckedou C07	KJ660347.1	Guinea	2014
Related virus				
* Marburg marburgvirus*	Marburg virus Ravn	EF446131	Kenya	1987
* Reston ebolavirus*	Reston virus	FJ621585	Philippines	2008
* Sudan ebolavirus*	Sudan virus - Boniface	AY729654	Uganda	2000

### Ebola RPA assay

Primers and probes were synthesized by Integrated DNA Technology (Iowa, USA), and purified by standard desalting. Assays were first optimised using synthetic gene fragments of one of the isolates (Accession number; KJ660348.1), from 470 to 2210 bp (1794 bp) as a template (data not shown). Optimal primers EBZ#3F (5’ TCT CGT CCT CAG AAA GTC TGG ATG ACG CCG) and EBZ#3R (5’ Biotin-TAC TTG ATA CAC TGG GAT GAC TCT TTG CCG) and probe EBZ#3P (5’ FAM-CTY ACT GAA TCT GAC ATG GAT TAC CAC AAG ATC /idSp/TR ACA GCA GGT CTG TCC /3SpC3/) amplified a 132 base pair fragment of the Ebolavirus Makona (Guinea) strain (Accession number KJ660348.1, amplification occurred between nucleotides 476 and 608). The Ebola RPA assay was performed using the TwistAmp™ nfo kit in pellet format (TwistDx, Cambridge, United Kingdom) according to manufacturer’s instructions, but with modification to the primers and probe concentrations and final volume. Briefly, 29.5 μL of rehydration buffer was mixed with 2.1 μL of each forward and reverse primer (10 μM) and 0.6 μL of the target specific probe (10 μM). Then 34.3 μL of this master mix was added to the dry reagent pellet, followed by 5 μL of the template, and the pellet resuspended by aspirating and dispensing several times. The reaction was activated by addition of 2.5 μL magnesium acetate (280 mM) to the reaction mix, followed by incubation at 37 °C for 30 min. After amplification, 1 μL of the amplified product was diluted with 9 μL of running buffer (Milenia Biotec, Giessen, Germany) and added to the sample pad of the HybriDetect lateral flow strip (Milenia Biotec, Giessen, Germany). Strips were placed into tubes containing 100 μL of running buffer for 5 min, and photographed using a digital camera. Grey-scale converted images were analysed using ImageJ software (National Institutes of Health, MD, USA) to determine band-intensity, by measuring the mean grey value (limit to threshold), using a fixed area measurement, and subtracting from the maximum grey value (255). For each test band, the average of two neighbouring white spaces was subtracted from the band intensity to normalize the results. A sample was defined as positive if the normalised band intensity was 1.3 times higher than the standard deviation of the two neighbouring white space values.

### Determination of analytical sensitivity and specificity

Following similar viral RNA assay development studies that employed RPA technology in establishing limit of detection using cloned gene [34, 35], we also performed standard ten-fold dilutions of an in-vitro synthesis (Genscript, USA) of the *Zaire ebolavirus* NP gene (Kikwit isolate) cloned into *EcoRI*/*XhoI* sites of pCAGGS. These pCAGGS were tested in two replicates using real-time TaqMan PCR with previously described Ebolavirus species specific primers and probe [43]. The assay was performed using SuperScript III Platinum Taq One-Step quantitative PCR Kit (Thermo Fisher Scientific, MA, USA) and the ABI7500 in Fast Mode. The plasmid dilutions were subsequently tested with RPA-LFS for determination of analytical sensitivity. Related Ebolaviruses (Table 1) and Dengue 1–4, *Plasmodium falciparum*, West Nile virus were also tested with RPA-LFS for determination of the analytical specificity of the assay.

## Results

### Establishing the RPA-LFS assay

*Zaire ebolavirus* species specific RPA primers and probes were designed based on the published sequence of the Guinea strain (Accession number; KJ660348.1) with the use of PRIMER-BLAST [44], which generated 20 nucleotides that were extended manually to a longer 30 nucleotides to be more amenable for RPA reactions. The sequences of the selected primers and probes were compared with an alignment of the NP gene sequences of seven isolates of the virus (Accession number; AF086833.2, KC242785.1, KC242796.1, KC242800.1, KC242792.1, KJ660346.1, KJ660347.1) to confirm homology with thee strains, which represented the various human outbreaks between 1976 and 1995 and the 2014 outbreak [2, 45–47]. Labelled primers and probes were tested for RPA followed by LFS detection (*Zaire* RPA-LFS) using a synthetic Ebolavirus template, and tested for optimal reaction times between 5 to 40 min. The optimal reaction time at 37 °C was 30 min, plus 5 min incubation of the LFS in the running buffer. A visible red colour band at the control and test lines were observed for positive controls while the no template control (negative control) had only one band at the control region of the strip. The control band on both strips indicated a valid RPA-LFS assay.

### Analytical sensitivity and specificity of the RPA-LFS assay

**Fig. 1:**
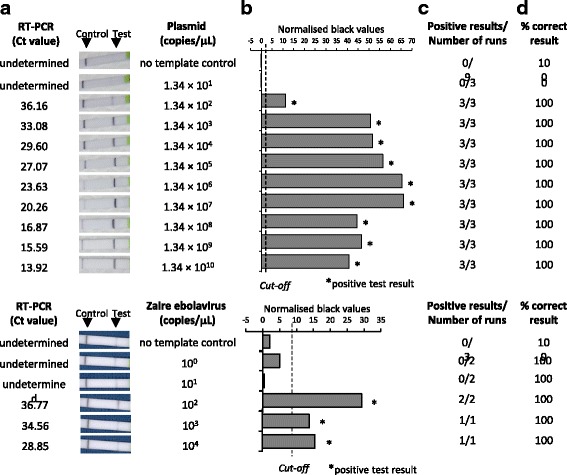
Analytical sensitivity of Ebolavirus RPA-LFS test. Sensitivity testing using both plasmid containing the Ebolavirus gene (top) and Zaire Ebolavirus RNA (bottom)**. a** RT-PCR cycle threshold (Ct) values for a single sample, along with corresponding photograph of LFS with control bands (all samples) and test bands (positive samples) compared to copy number of serially diluted template DNA or RNA (copies/μL) and no template control. **b** Normalised pixel density (black values) from the assay displayed in (**a**). **c** Positive results compared to number of runs tested at that dilution. **d** Analytical sensitivity displayed as percentage of correct results from all runs. The test line appeared at every dilution down to 10^2^ copies/μL, which was comparable with Real-Time PCR

**Fig. 2:**
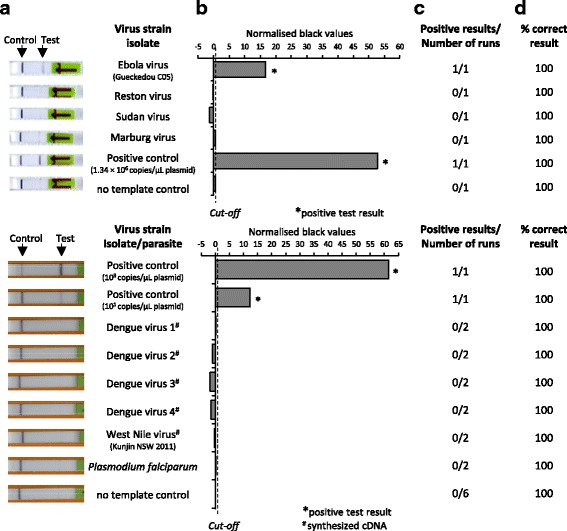
Specificity of the Ebolavirus RPA-LFS test. **a** Representative photograph of LFS with control bands and test bands of different viral isolates, *Plasmodium falciparum*, positive control genomic template DNA, or no template control. **b** Normalised pixel density (black values) from the test displayed in (**a**). **c** Positive results of all test runs compared to number of individual runs. **d** Specificity displayed as percentage of correct results from all runs

The detection threshold of the *Zaire* RPA-LFS was determined using a dilution series of plasmid containing the NP gene (1.34 × 10^10^ to 1.34 × 10^1^) copies / μL), and viral RNA from cultured Ebolavirus and comparing results to quantification using the gold standard real-time PCR assay. Detection using *Zaire* RPA-LFS showed the same sensitivity to RT-PCR, with a detection limit of 1.34 × 10^2^ copies / μL (Fig. 1) and was notably faster (30 min versus > 2 h). We note that a faint band was sometimes observed in no template negative controls, however, this faint band was clearly distinguishable from true positives, as demonstrated by ImageJ analysis of black pixel density. Such image analysis could be performed in field situations through the use of a lateral flow reader. We note that the faint test bands in the no template negative controls and Fig. 2 have been shown to disappear using higher dilutions of RPA product in the lateral flow device (e.g. 1:100–1:200) [48] compared to the 1:10 dilution that we used, however, this may affect sensitivity of the assay. We also observed that in the case of very high concentrations of hybridization product, the intensity of the control band was affected, but this did not affect interpretation of results.

**Fig. 3:**
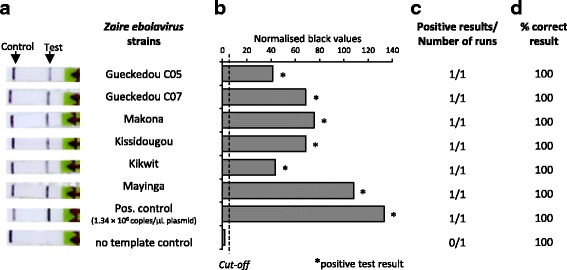
RPA-LFS detection of *Zaire ebolavirus* strains. **a** Photograph of LFS with control bands and test bands of different *Zaire ebolavirus* strains, positive control (1.34 × 10^6^ copies/μL synthetic template DNA) or no template control. **b** Normalised pixel density (black values) from the assay shown in (**a**). **c** Positive results compared to number of individual runs. **d** Specificity displayed as percentage of correct results

In order to determine cross reactivity or analytical specificity of our assay, we tested cultured viral RNA from Reston, Sudan and Marburg viruses, as well as different strains of *Zaire ebolavirus* species (Table 1). Marburg viruses show the same haemorrhagic symptoms as Ebolaviruses and Ebola cases were mistaken for Marburg virus when it was first discovered in 1976 [46]. We also tested *Plasmodium falciparum*, Dengue virus 1–4 and West Nile virus (Kunjin NSW 2011 strain), as these pathogens are likely to be isolated from clinical samples in the same geographic region. Our *Zaire ebolavirus* RPA-LFS did not show a positive test result when exposed to the *Marburg* RNA, any of the other *ebolavirus* species, or other pathogens (Fig. 2), indicating the assay was specific to *Zaire ebolavirus* species. We confirmed this specificity by demonstrating a test line consistently appeared when exposed to viral RNA from cultures of different *Zaire ebolavirus* strains (Fig. 3).

## Discussion

Reliable and rapid diagnosis of patients suspected of having EVD is critical to prevention, limiting the spread and management of treatment. This need was clearly made apparent in the Freetown declaration, which was a collective response to the Ebolavirus outbreak that killed more than 11,000 people, made by delegates from the African Society for Laboratory Medicine and the World Health Organisation in Freetown, Sierra Leone, in October 2015. The declaration included a strong call to build and strengthen laboratory networks to prevent, detect and respond to new and future diseases threats [7]. Importantly, innovative diagnostic tools that are comparable to current gold standards for resource-limited settings have been identified as critical to EVD diagnosis. In particular, a major challenge with RT-PCR is its inappropriateness for settings where there is lack of expertise and infrastructure to support its implementation. Typically, PCR facilities are many miles away from district hospitals where they are critically needed. Hence, suspected EVD samples sent to central facilities can take two or more days before the patients are aware of their status. This delay has major impacts on patient isolation, care and treatment.

In the very active space of Ebola diagnostics post the 2014 outbreak, the challenge still remains to develop a rapid, sensitive, selective Ebolavirus test, and to undergo full regulatory assessment and field testing. Tests for IgM/IgG [49, 50] cannot detect early-stages of the disease before patients have mounted an immune response. Antibody-based diagnostics detecting Ebolavirus antigens like ReEBOV Antigen Rapid Test (Corgenix, Broomfield, Colorado, USA); SD Q Line Ebola Zaire Ag test; (SD Biosensor, Suwon, Gyeonggi-do, Republic of Korea); Ebola (Senova Immunoassay Systems, Weimar, Germany); OraQuick Ebola Rapid Antigen Test (OraSure Technologies, Bethlehem, Pennsylvania, USA) are reasonably easy to operate and offer rapid on-site detection incorporating small quantities of blood or serum as material. However, these antibody based diagnostic methods are not as sensitive as their RT-PCR test counterparts. Thus, currently used reference assays solely detect viral RNA, such as the RealStar Filovirus Screen RT-PCR Kit 1.0 and its improved RealStar Zaire Ebolavirus RT-PCR Kit version, Altona Diagnostics. These RT-PCRs were originally designed for research only and hence mostly include labour-intensive RNA extraction steps, temperature-sensitive reagents, expensive laboratory equipment and the need for manual read-out. Therefore, highly trained staff are making multiple precision steps in sophisticated high containment laboratories to deliver an EVD result with a turnaround time from four to 6 h. In contrast, an automated system integrates sample purification, nucleic acid amplification and detection of the target sequence using RT-PCR, additionally lowering the risk for technical personnel. A verified example of an automated system is the GeneXpert® System combined with the Xpert® Ebola Assay (Cepheid, Sunnyvale, California, USA), able to deliver results in ~ 2.5 h. Other companies have also designed automated or semi-automated Ebola assays (Idylla™ EBOV test (Biocartis, Mechelen, Belgium); BioThreat/Ebola Panels (BioFire, Salt Lake City, Utah, USA) for point-of-care; these systems tend to be easily portable (7.8 to 25.2 kg) but come with a high price tag.

In contrast, here we report a molecular assay for *Zaire ebolavirus* species detection using a LFS, and only requiring simple and inexpensive equipment (such as a 37 °C heating block and lateral flow reader). The assay is simple, fast, comparable with the current gold standard, and reduces the infrastructural requirements and technical needs commonly associated with the RT-PCR-based assays. Our assay has the same detection limit as PCR (Fig. 1) and was highly specific for the *Zaire ebolavirus* strains, which have been responsible for different outbreaks since 1976 as well as the recent outbreak (Figs. 2 and 3). The assay employs a novel isothermal amplification technology, RPA, which has previously been applied for the detection of DNA and RNA organisms [31]. Our assay sensitivity and specificity are similar to previously published studies using RPA for the detection of RNA viruses [32–40].

RPA is yet to demonstrate its potential in the detection of nucleic acids directly from clinical samples, apart from urine [42]. For blood testing, it is anticipated that RNA extraction would need to be performed first, possibly using rapid methods that do not require centrifuges, such as the SpeedXtract Nucleic Acid Kit, with a 15 min extraction protocol and requires only 20 μL of blood serum (Qiagen Hilden, Germany) [51, 52]. This nucleic acid kit was deployed in combination with a prototype diagnostic that employs real time version of RPA during the recent Ebolavirus outbreak and is based on a magnetic particle technology that removes inhibitors [52]. Similarly, although we used a two-step reverse transcription approach in sample preparation, for field detection a one-step method could be used as demonstrated previously by RPA-detection of Rift Valley fever virus [35], where MuLV reverse transcriptase was included in the RPA reaction pellets, making the cDNA synthesis step easier and amenable to resource-limited settings. In addition, a disposable detection device that allows the assay to be performed in a closed system could be used to prevent cross-contamination of samples, contain the spread of amplified products, and protect the operator during the procedure [53]. We note that previous RPA assay publications have indicated a constant shaking speed is required for a more stable signal on the strip, and that agitation also improves sensitivity and amplification time, particularly when the detection limit is low [42]. However, our RPA was performed without agitation and yet achieved similar sensitivity to RT-PCR. We chose to ignore the shaking event, because in a typical routine diagnostic laboratory this step might be forgotten or omitted as a result of scheduled turnaround time with other routine diagnostic assays or work load. It is possible that with shaking our sensitivity may have been even more improved and could reduce the reaction time below 30 min.

Our RPA-LFS for *Zaire ebolavirus* species, as a qualitative assay, when combined with the technologies for rapid low-resource detection described above, could provide a rapid response to Ebolavirus outbreaks in the future. The cost of components is approximately USD $10 per test, without the sample preparation step. In the 2014/2015 Ebolavirus outbreak over 28,000 cases were diagnosed. If another epidemic were to build with a similar scale, it is critical to take space limitations in high-containment facilities into consideration. Therefore, complete inactivation of Ebolavirus samples is essential to allow specimens to be manipulated outside of biological contained facilities. Currently, our test system requires RNA extraction with a commercially available kit. However, this step may be easily replaced by the use of TRIzol® LS reagent (Invitrogen Corp., Carlsbad, CA), which successfully inactivates members of the Filovirus species [54]. This would enable the assay to be performed outside of a high containment facility, accelerating turnaround time to diagnose an EVD case. However, replacing the labour-intensive low-throughput RNA isolation with either a safe, low-cost, high-throughput version, or eliminating RNA extraction completely, is still highly desirable. Such an optimised Ebolavirus test version could have high potential to be compatible with unprocessed clinical material, such as whole blood obtained by finger-stick. Indeed, Clancy and colleagues (2015) have successfully shown the detection of *Streptococcus pneumoniae* with whole blood as material using an isothermal amplification approach by RPA [55]. A field trial of our Ebolavirus test or an optimized version during an Ebolavirus outbreak is essential. Yet, it is impossible to predict the strain that future outbreaks may be caused by. Our primers and probe may also detect these future strains because the genetic diversity within the Ebolavirus (particularly *Zaire ebolavirus* species) has been very low to date, with a maximum of 2.7% nucleotide difference between sequences [56]. This minimal diversity is also evident with the Guinea strain which showed 97% identity with previous strains [2]. Additionally, the designed oligonucleotides that utilized the Guinea strain, detected previous strains, demonstrating a versatility that may help detect future strains. Similar assays could be designed to detect other Filovirus species responsible for outbreaks.

## Conclusion

We have developed an Ebolavirus assay that could be implemented in low-resource laboratories that do not have the capacity for RT-PCR, and this assay could potentially be helpful in the next outbreak. However, field evaluation of the assay in a typical clinical setting will help to determine clinical sensitivity and specificity respectively, and areas of improvement.

## Abbreviations

ELISA: Enzyme-linked immunosorbent assay
EVD: Ebolavirus diseases
LFS: Lateral flow strip
PCR: Polymerase chain reaction
RPA: Recombinase polymerase amplification
RT: Real-time

## References

[1] World Health Organization (2015). Ebola situation report.

[2] Baize S, Pannetier D, Oestereich L (2014). Emergence of Zaire Ebola virus disease in Guinea. N Engl J Med.

[3] Lyon GM, Mehta AK, Varkey JB (2014). Clinical care of two patients with Ebola virus disease in the United States. N Engl J Med.

[4] Parra JM, Salmeron OJ, Velasco M (2014). The first case of Ebola virus disease acquired outside Africa. N Engl J Med.

[5] World Health Organization (2015). WHO commends Sierra Leone for stopping Ebola virus transmission.

[6] Funk S, Piot P (2014). Mapping Ebola in wild animals for better disease control. elife.

[7] African Society of Laboratory Medicine. The Freetown Declaration on Developing Resilient Laboratory Networks for the Global Health Security Agenda in Africa. Available at: https://aslm.org/what-we-do/global-health-security/freetown-declaration/. Accessed 10 Jan 2018.

[8] World Health Organization. Declaration of the end of Ebola virus disease outbreak in the Democratic Republic of the Congo. http://apps.who.int/iris/bitstream/10665/255798/1/EbolaDRC-02072017.pdf. Accessed 10 Jan 2018.

[9] Drosten C, Gottig S, Schilling S (2002). Rapid detection and quantification of RNA of Ebola and Marburg viruses, Lassa virus, Crimean-Congo hemorrhagic fever virus, Rift Valley fever virus, dengue virus, and yellow fever virus by real-time reverse transcription-PCR. J Clin Microbiol.

[10] Ksiazek TG, Rollin PE, Williams AJ (1999). Clinical virology of Ebola hemorrhagic fever (EHF): virus, virus antigen, and IgG and IgM antibody findings among EHF patients in Kikwit, Democratic Republic of the Congo, 1995. J Infect Dis.

[11] Leroy EM, Baize S, Lu CY (2000). Diagnosis of Ebola haemorrhagic fever by RT-PCR in an epidemic setting. J Med Virol.

[12] Panning M, Laue T, Olschlager S (2007). Diagnostic reverse-transcription polymerase chain reaction kit for filoviruses based on the strain collections of all European biosafety level 4 laboratories. J Infect Dis.

[13] Towner JS, Rollin PE, Bausch DG (2004). Rapid diagnosis of Ebola hemorrhagic fever by reverse transcription-PCR in an outbreak setting and assessment of patient viral load as a predictor of outcome. J Virol.

[14] Niikura M, Ikegami T, Saijo M, Kurane I, Miranda ME, Morikawa S (2001). Detection of Ebola viral antigen by enzyme-linked immunosorbent assay using a novel monoclonal antibody to nucleoprotein. J Clin Microbiol.

[15] Ksiazek TG, West CP, Rollin PE, Jahrling PB, Peters CJ (1999). ELISA for the detection of antibodies to Ebola viruses. J Infect Dis.

[16] Saijo M, Niikura M, Ikegami T, Kurane I, Kurata T, Morikawa S (2006). Laboratory diagnostic systems for Ebola and Marburg hemorrhagic fevers developed with recombinant proteins. Clin Vaccine Immunol.

[17] Vogel G (2014). Testing new Ebola tests. Science.

[18] The Conversation. New bedside test predicts Ebola infection in minutes, 2015. Available at: https://theconversation.com/new-bedside-test-predicts-ebola-infection-in-minutes-43836. Accessed 10 Jan 2018.

[19] German Primate Centre. Suitcase laboratory for rapid detection of Ebola, 2015. Available at: http://www.dpz.eu/en/news/news/single-view/news/entwickelt-labor-im-koffer-gegen-ebola-in-afrika.html. Accessed 10 Jan 2018.

[20] Poje JE, Kastratovic T, Andrew R, Ana M, Guillermo C, Troetti SE, Jabado OJ, Leigh Fanning M, Stefanovic D, Macdonald J (2014). Visual displays that directly Interface and provide read-outs of molecular states via molecular graphics processing units. Angew Chem Int Ed.

[21] Compton J (1991). Nucleic acid sequence-based amplification. Nature.

[22] Walker GT, Little MC, Nadeau JG, Shank DD (1992). Isothermal in vitro amplification of DNA by a restriction enzyme/DNA polymerase system. Proc Nat Acad Sci.

[23] Dean FB, Nelson JR, Giesler TL, Lasken RS (2011). Rapid amplification of plasmid and phage DNA using Phi29 DNA polymerase and multiply-primed rolling circle amplification. Genome Res.

[24] Notomi T, Okayama H, Masubuchi H, Yonekawa T, Watanabe K, Amino N, Hase T (2000). Loop mediated isothermal amplification of DNA. Nucleic Acids Res.

[25] Vincent M, Xu Y, Kong H (2004). Helicase-dependent isothermal DNA amplification. EMBO Rep.

[26] Fire A, Xu S (1995). Rolling replication of short DNA circles. Proc Natl Acad Sci.

[27] Wharam SD, Marsh P, Lloyd JS, Ray TD, Mock GA, Assenberg R, McPhee JE, Brown P, Weston A, Cardy DL (2001). Specific detection of DNA and RNA targets using a novel isothermal nucleic acid amplification assay based on the formation of a three-way junction structure. Nucleic Acids Res.

[28] Piepenburg O, Williams CH, Stemple DL, Armes NA (2006). DNA detection using recombination proteins. PLoS Biol.

[29] Guatelli JC, Whitfield KM, Kwoh DY, Barringer KJ, Richman DD, Gingeras TR (1990). Isothermal, in vitro amplification of nucleic acids by a multi enzyme reaction modeled after retroviral replication. Proc Natl Acad Sci.

[30] Zhang DY, Brandwein M, Hsuih TC, Li H (1998). Amplification of target-specific, ligation-dependent circular probe. Gene.

[31] James A, Macdonald J (2015). Recombinase polymerase amplification: emergence as a critical molecular technology for rapid, low-resource diagnostics. Expert Rev Mol Diagn.

[32] Crannell ZA, Rohrman B, Richards-Kortum R (2014). Equipment-free incubation of recombinase polymerase amplification reactions using body heat. PLoS One.

[33] Euler M, Wang Y, Otto P, Tomaso H, Escudero R, Anda P, Hufert FT, Weidmanna M (2012). Recombinase polymerase amplification assay for rapid detection of Franscisella tularensis. J Clin Microbiol.

[34] Abd El Wahed A, Patel P, Heidenreich D, Hufert FT, Weidmann M (2013). Reverse transcription recombinase polymerase amplification assay for the detection of middle east respiratory syndrome coronavirus. PLoS Curr.

[35] Eulera M, Wang Y, Nentwich O, Piepenburg O, Huferta FT, Weidmanna M (2012). Recombinase polymerase amplification assay for rapid detection of Rift Valley fever virus. J Clin Virol.

[36] Abd El Wahed A, El-Deeb A, El-Tholoth M, Abd El Kader H, Ahmed A (2013). A portable reverse transcription recombinase polymerase amplification assay for rapid detection of foot-and-mouth disease virus. PLoS One.

[37] Amera HM, Abd El Wahed A, Shalaby MA, Almajhdia FN, Hufert FT, Weidmann M (2013). A new approach for diagnosis of bovine coronavirus using a reverse transcription recombinase polymerase amplification assay. J Virol Methods.

[38] Mekuria TA, Zhang S, Eastwella KC (2014). Rapid and sensitive detection of little cherry virus 2 using isothermal reverse transcription-recombinase polymerase amplification. J Virol Methods.

[39] Euler M, Wang Y, Heidenreich D, Patel P, Strohmeier O, Hakenberg S, Niedrig M, Hufert FT, Weidmanna M (2013). Development of a panel of recombinase polymerase amplification assays for detection of biothreat agents. J Clin Microbiol.

[40] Aebischer A, Wernike K, Hoffmann B, Beer M (2014). Rapid genome detection of Schmallenberg virus and bovine viral diarrhea virus by use of isothermal amplification methods and high speed real-time reverse transcriptase PCR. J Clin Microbiol.

[41] Escadafal C, Faye O, Sall AA, Faye O, Weidmann M (2014). Rapid molecular assays for the detection of yellow fever virus in low-resource settings. PLoS Negl Trop Dis.

[42] Katrin K, Jekaterina F, Oana T (2014). Sensitive and rapid detection of chlamydia trachomatis by recombinase polymerase amplification directly from urine samples. J432 Mol Diagn.

[43] Trombley AR, Wachter L, Garrison J (2010). Short report: comprehensive panel of real-time TaqMan™ polymerase chain reaction assays for detection and absolute quantification of filoviruses, arenaviruses, and new world hantaviruses. Am J Trop Med Hyg.

[44] Ye J, Coulouris G, Zaretskaya I, Cutcutache I, Rozen S, Madden TL (2012). Primer-BLAST: a tool to design target-specific primers for polymerase chain reaction. BMC Bioinformatics.

[45] Report of an International Commission (1976). Ebola haemorrhagic fever in Zaire. Bull World Health Organ.

[46] Report of a WHO/International Study Team (1976). Ebola haemorrhagic fever in Sudan. Bull World Health Organ.

[47] Carroll SA, Towner SJ, Sealy KT (2013). Molecular evolution of viruses of the family Filoviridae based on 97 whole-genome sequences. J Virol.

[48] Saldarriaga OA, Castellanos-Gonzalez A, Porrozzi R, Baldeviano GC, Lescano AG, de Los Santos MB (2016). An innovative field-applicable molecular test to diagnose cutaneous Leishmania Viannia spp. infections. PLoS Negl Trop Dis.

[49] Lucht A, Formenty P, Feldmann H, Gotz M, Leroy E (2007). Development of an immunofiltration-based antigen-detection assay for rapid diagnosis of Ebola virus infection. J Infect Dis.

[50] Nakayama E, Yokoyama A, Miyamoto H, Igarashi M (2010). Enzyme-linked immunosorbent assay for detection of filovirus species-specific antibodies. Clin Vaccine Immunol.

[51] LabMedica. Rapid Ebola Virus Detection Assay Evaluated, 2015. Available at: http://www.labmedica.com/microbiology/articles/294761366/rapid-ebola-virus-detection-assay-evaluated.html. Accessed 10 Jan 2018.

[52] Faye O, Faye O, Soropogui B, Patel P, El Wahed AA, Loucoubar C, Fall G, Kiory D, Magassouba N’F, Keita S, Kondé MK, Diallo AA, Koivogui L, Karlberg H, Mirazimi A, Nentwich O, Piepenburg O, Niedrig M, Weidmann M, Sall AA. Development and deployment of a rapid recombinase polymerase amplification Ebola virus detection assay in Guinea in 2015. Euro Surveill. 2015;20(44). 10.2807/1560-7917.ES.2015.20.44.30053. 10.2807/1560-7917.ES.2015.20.44.30053 26558690

[53] Ustar Biotechnologies (Hangzhou) Ltd. Disposable Nucleic Acid Detection Device. Available at: http://www.bioustar.com/en/product_show.aspx?id=48. Accessed 10 Jan 2018.

[54] Blow JA, Dohm DJ, Negley DL, Mores CN (2004). Virus inactivation by nucleic acid extraction reagents. J Virol Methods.

[55] Clancy E, Higgins O, Forrest MS, Boo TW (2015). Development of a rapid recombinase polymerase amplification assay for the detection of streptococcus pneumoniae in whole blood. BMC Infect Dis.

[56] Carroll SA, Towner JS, Sealy TK, McMullan LK, Khristova ML, Burt FJ, Swanepoel R, Rollin PE, Nichola ST (2013). Molecular evolution of viruses of the family Filoviridae based on 97 whole-genome sequences. J Virol.

